# Establishment of a quality evaluation system of sweet potato starch using multivariate statistics

**DOI:** 10.3389/fnut.2022.1025061

**Published:** 2022-10-18

**Authors:** Chen Ma, Yi Zhang, Ruixue Yue, Wenting Zhang, Jian Sun, Zhimin Ma, Fuxiang Niu, Hong Zhu, Yunfeng Liu

**Affiliations:** ^1^Xuzhou Institute of Agricultural Sciences in Jiangsu Xuhuai District, Xuzhou, China; ^2^Institute of Cereal and Oil Crops, Hebei Academy of Agricultural and Forestry Sciences, Shijiazhuang, China; ^3^Hebei Zhongshu Agricultural Technology Group Co., Ltd., Qinhuangdao, China

**Keywords:** sweet potato starch, principal component analysis, analytic hierarchy process, quality evaluation system, evaluation model

## Abstract

**Background:**

The quality of starch greatly affects the quality of processed products. There are many indexes for quality evaluation of starch. Currently, amylose content is considered the chief index in the quality evaluation of sweet potato starch, which is entirely based on tradition (experience) method. The existing evaluation standards lack quality evaluation standards for sweet potato starch.

**Purpose:**

To screen reasonable evaluation indexes of sweet potato starch, and establish a scientific and systematic evaluation system of sweet potato starch.

**Methods:**

Twenty-two components and quality indexes of sweet potato starch were measured. The evaluation indexes of sweet potato starch were screened based on a statistical description, correlation analysis, and principal component analysis (PCA), and a quality evaluation model of sweet potato starch for brewing was established based on analytic hierarchy process. The calculated values of the model were verified by linear fitting with standardized sensory evaluation values.

**Results:**

The coefficient of variation of total starch content (%), amylose content (%), amylopectin content (%), L* value, Δ*E*, water absorption capacity (g/g), and pasting temperature was less than 6%, while the coefficient of variation of other indexes was larger. In addition, there were different degrees of correlation among the indexes. PCA was used to identify interrelated variables, and the first six principal components together account for 82.26% of the total variability. Then, seven core indexes — setback (cp), rate of regression (%), ratio of amylose to amylopectin (%), gel strength (kgf/cm^2^), a* value, ash content (%), and solubility (%) — were selected from the six principal components according to the load value of the rotation matrix. These seven core indexes replaced the original 22 indexes to simplify the evaluation of sweet potato starch. The quality evaluation model of sweet potato starch was *Y* = 0.034X_2_ + 0.321X_6_ + 0.141X_8_ + 0.08X_17_ + 0.023X_19_ + 0.08X_21_ + 0.321X_22_.

**Conclusion:**

The comprehensive evaluation system of sweet potato starch can accurately predict the quality of sweet potato starch. The development of such a system is of great significance to the post-harvest processing of high-starch sweet potato and the breeding of high-quality and high-starch sweet potato varieties.

## Introduction

Starch, as a storage nutrient for plants, is the main nutrient needed for human energy. It is found in cereals, roots, stems, and seeds, and it is widely used in foods, textiles, paper, materials, and pharmaceuticals (1, 2). Starch mainly exists in plants in the form of starch granules. Natural starch granules are composed of an inner amylose layer and an outer amylopectin layer. Both amylose and amylopectin are composed of D-glucose. Amylose is a linear polysaccharide linked by α(1–4)-glycosidic bonds, whereas amylopectin is a multi-branched polymer linked by α(1–4)-glycosidic bonds and α(1–6)-glycosidic bonds (3). The composition, structure, arrangement, and crystallization of starch from different sources results in different functional, processing, and quality characteristics. In recent years, researchers have focused on the relationship between starch fine structure and physicochemical properties. Tong et al. (4) found that the pasting and texture properties were affected by the fine structure of both amylose and amylopectin. The pasting and thermal properties were influenced by the debranched starch fine structure of amylose and amylopectin, while the texture properties were mainly influenced by amylopectin fine structure. However, the quality characteristics of starch and their effect on processing suitability and quality are not clear yet. Currently, only the relationship between the structure and physicochemical properties of starch and starch quality has been analyzed. Moreover, there is no systematic quantitative evaluation of starch quality. Han et al. (5) found that when the short-chain content of amylopectin in rice was high, the molecular chain was loose, resulting in unstable starch that readily expanded and was difficult to rearrange. In addition, the pasting time, pasting temperature, and setback values were small. As a result, the rice was susceptible to pasting and retrogradation (5).

Sweet potato, an annual or perennial root crop of the Convolvulaceae family, is the seventh most important food crop in the world (6, 7). Sweet potato has rich nutritional value, such as high levels of starch, carotene, dietary fiber, and more than 10 types of mineral elements (8, 9). Sweet potato starch is mainly composed of amylopectin, with a content of 68.8–86.79% (10, 11). There are negatively charged phosphate ester groups in the starch particles of sweet potato, which afford good swelling ability. It has been widely used in food production fields, such as in brewing starch paste, as well as in making starch noodles. In addition, it can be used as a thickener, stabilizer, and water retention agent to improve the viscosity, water retention capacity, and water holding capacity of certain food systems (12, 13). Different varieties of sweet potato starch have different components and fine structures, resulting in different functions and processing suitability of starch. Therefore, it is urgently needed to establish the quality evaluation standard for sweet potato starch in post-harvest processing of high-starch sweet potato. Currently, amylose content is considered the chief standard in the quality evaluation of sweet potato starch. As reported by Tan et al., Sun et al., and Xing et al., during gelatinization, starch with high amylose content had high viscosity and was easy aging, and quality of starch noodle was good, so the amylose content can be used as an index to evaluate the quality of starch (11, 14, 15). This evaluation method is entirely based on tradition (experience) method and neglects many influencing indexes. Thus, starch quality cannot be accurately predicted. At the same time, the existing evaluation standards lack quality evaluation standards for special sweet potato starch. Therefore, it is very important to select appropriate evaluation indexes and establish a simple, efficient, and accurate comprehensive evaluation system for sweet potato starch.

In particular, brewing sweet potato starch is a form of sweet potato starch that is popular in China, it can be cooked as sweet potato starch thick soup. Currently, there is no systematic quality evaluation system for brewing sweet potato starch. Therefore, using evaluation indexes to describe the composition, physicochemical properties, and processing parameters, a systematic and comprehensive quality evaluation system was constructed for sweet potato starch. Specifically, this study obtained the quality evaluation index data of starch from eighteen sweet potato varieties. Next, through statistical description, correlation analysis, principal component analysis (PCA), and analytic hierarchy process (AHP), the core evaluation indexes were selected, and the quality evaluation system of brewing sweet potato starch was established. Finally, the effectiveness of the comprehensive quality evaluation model was verified by sensory evaluation results.

## Materials and methods

### Materials

Eighteen varieties of sweet potato, namely Yanshu 29, Yushu 15, Wanshu 9, Luxuan 1, Jishu 25, Qinshu 9, Jishu 99, Xushu 18, Xushu 37, Sushu 18, Sushu 23, Sushu 28, Luoxushu 9, Luoshu 10, Luoshu 11, Luoshu 12, Luoshu 14, and Luoshu 15, were harvested in the experimental field of the Sweet Potato Research Institute of the Chinese Academy of Agricultural Sciences (34° 16′N, 117° 17′E) on October 21, 2020. After callus treatment at high temperature, sweet potato roots of uniform size with no insect infestation or scars were selected for further treatment.

### Isolation of sweet potato starches

The starch was extracted using the method reported by Vithu et al., with slight modification (16). Clean sweet potato cubes (500 g) and water (1000 g) were mixed and homogenized thoroughly. The homogenate was filtered with four layers of gauze, passed through a 100-mesh sieve, and washed twice. The resultant slurry settled naturally over 12 h. The obtained precipitate was washed once more with water. Finally, the resultant starch was dried at 50°C for 24 h, ground into a powder, and passed through a 100-mesh sieve. Each variety was analyzed in triplicate.

### Composition measurement

The moisture content and ash content were determined according to the AOAC method (17). The contents of total starch, and amylose were determined with respective kits. The ratio of amylose to amylopectin was denoted as AA (18).

### Determination of color

The color of sweet potato starch was determined by a spectrophotometric colorimeter (CM-5, KONICA MINOLTA, Tokyo, Japan). The measurement indexes were L*, a*, b*, and Δ*E*, where L* represents the sample brightness; a* represents the red and green value; b* represents the yellow and blue value; and Δ*E* represents the total chroma.

### Water absorption capacity and oil absorption capacity

For water absorption capacity (WAC), 0.6 g of starch was weighed in a 50 mL centrifuge tube, and 10 mL of distilled water was added. The sample mixture was placed in a water bath at 30°C for 30 min and was centrifuged (3000 *g*, 15 min). Then, the water was removed. The centrifuge tube was inverted for 2 min and weighed. WAC (g/g) was calculated using Eq. 1. For oil absorption capacity (OAC), 1 g of starch was weighed in a 10 mL centrifuge tube, and 6 mL of soybean oil was added. The sample mixture was placed in a water bath at 30°C for 30 min and was centrifuged (3000 *g*, 15 min). The oil was removed. The centrifuge tube was inverted for 15 min and weighed. OAC (g/g) was calculated using Eq. 2 (19):


WAC(g/g) =

(1) $$\frac{\begin{array}{c} \mathrm{Weight}\,\mathrm{of}\,\mathrm{tube}\,\mathrm{after}\,\mathrm{removal}\,\mathrm{of}\,\mathrm{excess}\,\mathrm{water}-\mathrm{Weight}\,\mathrm{of}\,\mathrm{tube}\\ -\mathrm{sample}\,\mathrm{weight} \end{array}}{\mathrm{Sample}\,\mathrm{weight}}$$

OAC(g/g) =

(2) $$\frac{\begin{array}{c} \mathrm{Weight}\,\mathrm{of}\,\mathrm{tube}\,\mathrm{after}\,\mathrm{removal}\,\mathrm{of}\,\mathrm{excess}\,\mathrm{oil}-\mathrm{Weight}\,\mathrm{of}\,\mathrm{tube}\\ -\mathrm{sample}\,\mathrm{weight} \end{array}}{\mathrm{Sample}\,\mathrm{weight}}$$

### Pasting properties

Pasting properties were measured using a rapid viscosity analyzer (RVA) (4500, Perten Instruments, Stockholm, Sweden), according to a method reported by Qian et al. (20). Three grams of starch and 25 mL of distilled water were transferred to an RVA pot, stirred well, and heated using a temperature gradient. The sample mixture was equilibrated at 50°C for 1 min and heated to 95°C at a rate of 11.25°C/min. The mixture was kept at 95°C for 4.5 min, cooled to 50°C at 11.25°C/min, and held at 50°C for 3.5 min. The speed of the paddle was 160 r/min. In addition to the peak viscosity (PV), trough viscosity (TV), and final viscosity (FV), RVA characteristic parameters were also described by breakdown (BD = PV-TV), setback (SB = FV-TV), and pasting temperature (PT).

### Swelling power and solubility

In a 50 mL centrifuge tube, 0.4 g of starch was weighed, and 20 mL of distilled water was added. After shaking, the sample was placed in a water bath at 85°C for 30 min and centrifuged (6000 *g*, 15 min). The supernatant was collected, and the weight of the precipitate after starch expansion was measured. The supernatant was dried to a constant weight in a 105°C oven and weighed (21). The swelling power (SP) and solubility were calculated as shown in Eq. 3 and 4:


(3) $$\mathrm{SP}\left(\%\right)=\frac{\mathrm{Weight}\,\mathrm{of}\,\mathrm{precipitate}}{\mathrm{Sample}\,\mathrm{weight}\times \left(100-\mathrm{Solubility}\right)}\times 100$$

(4) $$\mathrm{Solubility}\left(\%\right)=\frac{\mathrm{Weight}\,\mathrm{of}\,\mathrm{supernatant}\,\mathrm{after}\,\mathrm{drying}}{\mathrm{Sample}\,\mathrm{weight}}\times 100.$$

### Rate of retrogradation

In a 50 mL centrifuge tube, 1.5 g of starch was weighed, and 25 mL of distilled water was added. After shaking, the sample was placed in a water bath at 95°C for 20 min, cooled at 25°C for 30 min, frozen at 4°C for 24 h, and thawed at 30°C for 2 h. Then, the sample was centrifuged at 3000 r/min for 20 min. Finally, the supernatant weight was weighed, and the rate of retrogradation (RR) was calculated (Eq. 5) (22):


(5) $$\mathrm{RR}\left(\%\right)=\frac{\mathrm{Supernatant}\,\mathrm{weight}}{\mathrm{Weight}\,\mathrm{of}\,\mathrm{the}\,\mathrm{starch}\,\mathrm{and}\,\mathrm{water}}\times 100.$$

### Gel strength

In a 100 mL beaker, 3 g of starch was weighed, and 50 mL of distilled water was added. The beaker containing the sample was placed in a water bath at 95°C for 30 min with constant agitation. Subsequently, it was cooled to room temperature, wrapped with plastic wrap, and placed in a refrigerator at 4°C for 24 h. After 24 h, the sample was taken out and balanced for 2 h at room temperature to form a stable starch gel. The gel strength (GS) of the starch was measured using a texture analyzer (TMS-PRO, FTC, Sterling, VA, United States). A cylinder probe with a diameter of 6 mm was selected. The pre-test speed was 2 mm/s, the test speed was 1 mm/s, and the post-test speed was 5 mm/s. The compression ratio was 50%. The triggering force was determined according to the instrument situation. During puncture, the GS was determined as the maximum stress per unit area of the probe (23).

### Standardization of evaluation indexes

There were different dimensions and orders of magnitude for the quality comprehensive evaluation indexes of sweet potato starch. Therefore, the normalization method was adopted to eliminate the influence of each index dimension on the results (24). The original data were initialized first, and the initialized value was the absolute value of the distance between each quality index value and the ideal value. Subsequently, the data were normalized using the normalized value X_*i*_ = $1-\frac{x^{\prime }\,\text{i}}{x^{\prime }\,\text{imax}}$, where x′_*i*_ is the initialization value, and x′_*imax*_ is the maximum initialization value.

### Determination of weight of evaluation index

To show the importance of different indexes in the comprehensive evaluation, a 1–9 scale method was used to determine the weight coefficients of each core index. Each index variable was set as *F*_*i*_, and the corresponding normalized value was X_*i*_. A judgment matrix ***A*** = (a_*ij*_) was established using the 1–9 scale method, and each element in the matrix was assigned using the following rules (25):

1) When *F*_*i*_ and *F*_*j*_ were equally important, a_*ij*_ = 1;
2) When *F*_*i*_ was slightly more important than *F*_*j*_, a_*ij*_ = 3;
3) When *F*_*i*_ was more important than *F*_*j*_, a_*ij*_ = 5;
4) When *F*_*i*_ was much more important than *F*_*j*_, a_*ij*_ = 7;
5) When *F*_*i*_ was extremely more important than *F*_*j*_, a_*ij*_ = 9; and
6) When between the above judgments, a_*ij*_ = 2, 4, 6, 8.

The weight *W*_*i*_ of each quality evaluation index was calculated according to the judgment matrix **A** (26).

1) Calculate the product of each row of the judgment matrix ***A***: *M*_*i*_ = $\prod_{\text{j}=1}^{\text{n}}\text{aij}$, i = 1, 2, …, n;
2) Calculate the n-root of *M*_*i*_: $\bar{W_{\text{i}}}=\sqrt[{\text{n}}]{M_{\text{i}}}$;
3) Calculate the *W*_*i*_: *W*_*i*_ = $\frac{\bar{w\,\text{i}}}{\sum_{\text{j}=1}^{\text{n}}\bar{w\,\text{j}}}$;
4) Calculate the maximum eigenvalue of the judgment matrix ***A***: $\lambda \,\max=\sum_{\text{j}=1}^{\text{n}}\frac{\left(A\,W\right)\,i}{\text{n}\,W\,\text{i}}$, where ***W*** = (*W*_1_, *W*_2_, …, *W*_*n*_)*^T^* is the eigenvector, and (***AW***)_*i*_ is the ith element of ***AW***;
5) Calculate the consistency index *C*_*I*_: *C*_*I*_ = $\frac{\lambda \,\max-\text{n}}{n-1}$. If the consistency ratio *C*_*R*_ = $\frac{C\,I}{R\,I}$ < 0.1 (where *R*_*I*_ is the mean random consistency index), the judgment matrix meets the consistency requirements; and
6) The synthetic value is obtained by the AHP: Y = $\sum_{j=1}^{n}W\,i\,X\,i$.

### Sensory evaluation and normalization of sweet potato starch

Ten trained sensory evaluators were organized to perform a sensory evaluation on sweet potato starch, and sensory data were normalized. The normalized value of sensory evaluation was Z_*i*_ = $\frac{z\text{i-}z\;\min}{z\;\max-z\;\min}$, where z_*i*_, z_*max*_, and z_*min*_ are the comprehensive score, highest score, and lowest score of sensory evaluation, respectively.

### Statistical analysis

SPSS 18.0 software was used for Variance analysis, and PCA. Duncan multiple comparison method was used to test the significance of Variance analysis (*P* < 0.05). Origin 2021 software was used for correlation analysis. Each experiment was analyzed in triplicate, and the experimental data were expressed as mean ± standard deviation (Mean ± SD).

## Results

### Composition of sweet potato starch

As shown in Table 1, the compositions of 18 kinds of sweet potato starch varied. The moisture content of different sweet potato starch was similar, with a range of 11.03–15.12% and a coefficient of variation of 9.26%. Ash content was significantly different, ranging from 0.40 to 1.68%, and the coefficient of variation was 32.44%. The result was consistent with the study of Rocha et al. (27).

**TABLE 1:** Composition of sweet potato starch (wet basis).

Variety	Moisture (%)	Ash (%)	Total starch (%)	Amylose (%)	Amylopectin (%)	AA (%)
Yanshu 29	11.53 ± 0.01^fg^	0.98 ± 0.15^bcdefg^	81.14 ± 5.61^abc^	20.84 ± 0.45^ab^	60.30 ± 6.06^abcd^	34.76 ± 4.24^a^
Yushu 15	11.49 ± 0.42^fg^	0.92 ± 0.46^defg^	85.90 ± 4.45^a^	19.23 ± 0.93^bcd^	66.67 ± 3.52^ab^	28.85 ± 0.13^bc^
Wanshu 9	11.27 ± 0.07^g^	1.44 ± 0.07^ab^	80.52 ± 3.24^abc^	19.27 ± 0.83^bcd^	61.26 ± 4.08^abcd^	31.57 ± 3.46^abc^
Luxuan 1	12.91 ± 0.01^bcde^	0.74 ± 0.07^efgh^	83.43 ± 3.86^ab^	21.65 ± 0.45^a^	61.78 ± 3.42^abcd^	35.08 ± 1.21^a^
Jishu 25	13.50 ± 0.32^bcd^	0.64 ± 0.07^fgh^	79.29 ± 1.58^abc^	20.57 ± 0.45^ab^	58.72 ± 1.13^bcd^	35.04 ± 0.10^a^
Qinshu 9	12.86 ± 0.21^bcde^	1.22 ± 0.33^abcd^	86.79 ± 4.03^a^	18.10 ± 0.17^d^	68.69 ± 4.20^a^	26.41 ± 1.86^c^
Jishu 99	12.00 ± 0.06^efg^	1.32 ± 0.18^abcd^	76.11 ± 3.24^bc^	19.94 ± 1.26^bc^	56.17 ± 1.99^cd^	35.48 ± 0.98^a^
Xushu 18	12.65 ± 0.09^cdef^	0.55 ± 0.07^gh^	80.52 ± 2.99^abc^	18.72 ± 0.32^cd^	61.81 ± 2.68^abcd^	30.30 ± 0.80^abc^
Xushu 37	13.05 ± 0.21^bcde^	1.19 ± 0.00^bcde^	73.29 ± 2.49^c^	17.79 ± 1.04^d^	55.50 ± 1.45^d^	32.03 ± 1.04^ab^
Sushu 18	11.34 ± 0.13^g^	0.94 ± 0.08^cdefg^	80.35 ± 1.75^abc^	17.78 ± 0.36^d^	62.56 ± 2.11^abcd^	28.45 ± 1.54^bc^
Sushu 23	11.27 ± 0.01^g^	0.91 ± 0.47^defg^	83.79 ± 4.11^ab^	20.46 ± 0.97^ab^	63.32 ± 5.09^abcd^	32.49 ± 4.15^ab^
Sushu 28	11.27 ± 0.01^g^	1.33 ± 0.22^abcd^	80.17 ± 3.82^abc^	20.48 ± 0.27^ab^	59.69 ± 4.09^bcd^	34.41 ± 2.82^a^
Luoxushu 9	12.29 ± 0.01^defg^	1.68 ± 0.05^a^	79.20 ± 1.12^abc^	20.58 ± 0.00^ab^	58.63 ± 1.12^bcd^	35.10 ± 0.68^a^
Luoshu 10	15.12 ± 0.06^a^	1.44 ± 0.07^ab^	83.52 ± 4.32^ab^	18.40 ± 0.60^cd^	65.12 ± 3.72^ab^	28.28 ± 0.69^bc^
Luoshu 11	11.03 ± 0.28^g^	1.02 ± 0.04^bcdef^	81.14 ± 4.11^abc^	19.71 ± 0.54^bc^	61.43 ± 4.66^abcd^	32.21 ± 3.33^ab^
Luoshu 12	13.58 ± 0.04^bc^	1.34 ± 0.06^abcd^	84.67 ± 2.61^ab^	19.96 ± 0.69^bc^	64.70 ± 1.92^abc^	30.85 ± 0.15^abc^
Luoshu 14	12.05 ± 0.14^efg^	1.41 ± 0.08^abc^	85.62 ± 2.82^a^	20.72 ± 0.50^ab^	64.90 ± 2.32^abc^	31.93 ± 0.37^ab^
Luoshu 15	14.06 ± 2.18^ab^	0.40 ± 0.14^h^	80.70 ± 4.49^abc^	19.31 ± 0.80^bcd^	61.39 ± 5.29^abcd^	31.63 ± 4.02^abc^
Maximum	15.12	1.68	86.79	21.65	68.69	35.48
Minimum	11.03	0.40	73.29	17.78	55.50	26.41
Range	4.10	1.29	13.49	3.87	13.18	9.08
Average	12.40	1.08	81.45	19.64	61.81	31.94
SD	1.15	0.35	3.43	1.14	3.44	2.73
Variation coefficient	9.26	32.44	4.21	5.79	5.57	8.53

M ± SD. For the same index, different letters indicate significant differences with *p* < 0.05. SD, standard deviation; AA, ratio of amylose to amylopectin.

The purity (total starch) of 18 kinds of sweet potato starch was similar and high, ranging from 73.29 to 86.79%, and the coefficient of variation was 4.21%. The amylose content and amylopectin content were significantly different, ranging from 17.78 to 21.65% and 55.50 to 68.69% respectively, and the coefficient of variation was 5.79 and 5.57%, respectively. The results showed that amylopectin was the main component of sweet potato starch, which was consistent with the study of Aina et al. (10). The amylose content of Luxuan 1 was the highest.

The larger the AA is, the higher the ratio of amylose to total starch is. The AA of 18 kinds of sweet potato starch was significantly different, ranging from 26.41 to 35.48% with a variation coefficient of 8.53%. AA of Jishu 99 was the highest, and amylose accounted for a large proportion of total starch.

### Color of sweet potato starch

Color is an intuitive index to evaluate the quality of starch, and it affects the quality of starch products, such as starch noodles (11, 28). The L* value and Δ*E* are two main indexes to evaluate starch color. As shown in Table 2, L* value and Δ*E* of Jishu 99 were the highest among the sweet potato varieties, and a* and b* values were small, indicating that the starch of Jishu 99 had the brightest and highest color quality. In contrast, L* value and Δ*E* of Xushu 37 were the lowest, and the absolute a* and b* values were the largest, indicating that the hue of the starch was reddish yellow and that its color was dull.

**TABLE 2:** Color of sweet potato starch.

Variety	L*	a*	b*	Δ*E*
Yanshu 29	96.22 ± 0.00^de^	–0.45 ± 0.16^kl^	3.03 ± 0.01^l^	96.27 ± 0.00^cd^
Yushu 15	96.25 ± 0.01^d^	–0.33 ± 0.01^ijk^	3.28 ± 0.00^i^	96.30 ± 0.01^c^
Wanshu 9	96.45 ± 0.17^bc^	–0.26 ± 0.01^hij^	2.82 ± 0.01^o^	96.49 ± 0.17^b^
Luxuan 1	95.86 ± 0.01^h^	0.14 ± 0.01^b^	3.33 ± 0.01^h^	95.91 ± 0.01^g^
Jishu 25	93.77 ± 0.01^m^	–0.49 ± 0.01^l^	3.27 ± 0.01^i^	93.82 ± 0.01^l^
Qinshu 9	96.08 ± 0.00^g^	–0.18 ± 0.00^efgh^	2.86 ± 0.01^n^	96.12 ± 0.00^f^
Jishu 99	96.57 ± 0.01^a^	–0.08 ± 0.28^def^	2.57 ± 0.01^p^	96.61 ± 0.01^a^
Xushu 18	96.50 ± 0.02^ab^	–0.25 ± 0.01^ghij^	3.73 ± 0.00^d^	96.58 ± 0.02^a^
Xushu 37	90.44 ± 0.01^n^	0.51 ± 0.00^a^	4.72 ± 0.01^a^	90.56 ± 0.01^m^
Sushu 18	94.98 ± 0.02^l^	0.08 ± 0.01^bc^	3.51 ± 0.01^e^	95.05 ± 0.02^k^
Sushu 23	96.42 ± 0.01^c^	–0.35 ± 0.01^jk^	3.12 ± 0.00^k^	96.47 ± 0.01^b^
Sushu 28	95.00 ± 0.02^l^	–0.28 ± 0.00^hij^	4.37 ± 0.01^b^	95.10 ± 0.02^k^
Luoxushu 9	95.58 ± 0.02^j^	–0.20 ± 0.00^fghi^	3.27 ± 0.01^i^	95.64 ± 0.02^i^
Luoshu 10	95.73 ± 0.01^i^	–0.05 ± 0.01^cde^	3.23 ± 0.01^j^	95.78 ± 0.01^h^
Luoshu 11	95.46 ± 0.01^k^	–0.52 ± 0.00^l^	4.22 ± 0.00^c^	95.56 ± 0.01^j^
Luoshu 12	96.13 ± 0.01^fg^	–0.12 ± 0.00^defg^	3.45 ± 0.01^f^	96.19 ± 0.01^ef^
Luoshu 14	96.06 ± 0.06^g^	0.01 ± 0.01^cd^	3.36 ± 0.01^g^	96.12 ± 0.06^f^
Luoshu 15	96.16 ± 0.01^ef^	–0.15 ± 0.01^efgh^	2.88 ± 0.01^m^	96.20 ± 0.01^de^
Maximum	96.57	0.51	4.72	96.61
Minimum	90.44	–0.52	2.57	90.56
Range	6.13	1.03	2.15	6.04
Average	95.54	–0.17	3.39	95.60
SD	1.45	0.25	0.56	1.43
Variation coefficient	1.52	151.54	16.57	1.50

M ± SD. For the same index, different letters indicate significant differences with *p* <0.05. SD, standard deviation.

### Pasting properties of sweet potato starch

Pasting temperature is the lowest temperature of gelatinized starch and reflected energy consumption. PV is the maximum viscosity after the suspension gelatinized with rising temperature, and it reflects the swelling power of starch particles. TV refers to the lowest viscosity value of starch from gel to collosol due to increased spacing between starch molecules. FV is the stable viscosity value after the solution changed from collosol to gel with decreasing temperature. BD reflects the shear resistance of starch, and SB reflects the short-term rearrangement capacity of starch (29). The pasting properties of all samples are presented in Table 3. The PV, TV, BD, FV, SB, and PT of 18 sweet potato starches were significantly different and ranged from 2294–3579 cp, 1721.5–2214 cp, 476.5–1365 cp, 2404.5–3361 cp, 683–1252.5 cp, and 75.08–81.55°C, respectively. This result is consistent with that of other researchers (20, 30). The PT of Sushu 28 was the lowest (75.08 ± 0.04°C), while that of Jishu 99 was the highest (81.55 ± 0.00°C) (Table 3). In the gelatinization process, different varieties of sweet potato starch had different energy consumption, which depended on the interaction between starch molecules. The BD of Luoshu 10 was the lowest, indicating that Luoshu 10 was more stable among the sweet potato varieties. The SB of Yushu 15 was the lowest, indicating that Yushu 15was not easy to retrogradation in the measured range.

**TABLE 3:** The pasting parameters of sweet potato starch.

Variety	PV (cp)	TV (cp)	BD (cp)	FV (cp)	SB (cp)	PT (°C)
Yanshu 29	2825.00 ± 41.01^fg^	1858.50 ± 41.72^e^	966.50 ± 0.71^d^	2671.50 ± 48.79^fg^	813.00 ± 7.07^fg^	77.15 ± 0.57^ef^
Yushu 15	2686.00 ± 39.60^ghi^	1721.50 ± 7.78^f^	964.50 ± 31.82^d^	2404.50 ± 26.16^h^	683.00 ± 18.38^i^	77.58 ± 0.04^de^
Wanshu 9	2627.00 ± 91.92^i^	1897.00 ± 28.28^de^	730.00 ± 63.64^g^	2635.50 ± 36.06^fg^	738.50 ± 7.78^h^	77.95 ± 0.64^cde^
Luxuan 1	3114.00 ± 25.46^cd^	2007.50 ± 45.96^bcd^	1106.50 ± 20.51^c^	2750.50 ± 79.90^fg^	743.00 ± 33.94^h^	77.93 ± 0.53^cde^
Jishu 25	3326.00 ± 41.01^b^	2080.00 ± 31.11^b^	1246.00 ± 9.90^b^	3172.50 ± 48.79^b^	1092.50 ± 17.68^b^	77.15 ± 0.49^ef^
Qinshu 9	2659.00 ± 98.99^hi^	1904.50 ± 28.99^de^	754.50 ± 70.00^fg^	2690.50 ± 48.79^fg^	786.00 ± 19.80^gh^	77.13 ± 0.60^ef^
Jishu 99	2747.50 ± 74.25^ghi^	1924.00 ± 56.57^cde^	823.50 ± 17.68^ef^	2734.50 ± 65.76^fg^	810.50 ± 9.19^fg^	81.55 ± 0.00^a^
Xushu 18	2807.50 ± 21.92^fgh^	2067.50 ± 41.72^b^	740.00 ± 19.80^g^	2935.00 ± 56.57^d^	867.50 ± 14.85^de^	77.88 ± 0.60^cde^
Xushu 37	3579.00 ± 80.61^a^	2214.00 ± 38.18^a^	1365.00 ± 42.43^a^	3304.00 ± 50.91^a^	1090.00 ± 12.73^b^	78.35 ± 0.00^bcde^
Sushu 18	2671.50 ± 86.97^ghi^	1895.50 ± 43.13^de^	776.00 ± 43.84^fg^	2770.00 ± 74.95^ef^	874.50 ± 31.82^de^	75.43 ± 0.60^g^
Sushu 23	2999.50 ± 62.93^de^	2002.00 ± 65.05^bcd^	997.50 ± 2.12^d^	2888.00 ± 35.36^de^	886.00 ± 29.70^de^	79.55 ± 0.49^b^
Sushu 28	3345.50 ± 44.55^b^	2208.50 ± 60.10^a^	1137.00 ± 15.56^c^	3361.00 ± 65.05^a^	1152.50 ± 4.95^a^	75.08 ± 0.04^g^
Luoxushu 9	2761.00 ± 72.12^ghi^	2041.50 ± 82.73^b^	719.50 ± 10.61^g^	3099.00 ± 38.18^bc^	1057.50 ± 44.55^b^	77.10 ± 0.49^ef^
Luoshu 10	2294.00 ± 73.54^j^	1817.50 ± 57.28^ef^	476.50 ± 16.26^i^	2617.00 ± 86.27^g^	799.50 ± 28.99^fg^	79.10 ± 0.07^bc^
Luoshu 11	3168.00 ± 65.05^c^	2025.00 ± 60.81^bc^	1143.00 ± 4.24^c^	2999.00 ± 33.94^cd^	974.00 ± 26.87^c^	78.70 ± 0.64^bcd^
Luoshu 12	2700.50 ± 72.83^ghi^	2068.50 ± 37.48^b^	632.00 ± 35.36^h^	2956.00 ± 57.98^d^	887.50 ± 20.51^de^	76.28 ± 0.67^fg^
Luoshu 14	2631.00 ± 73.54^i^	1992.50 ± 37.48^bcd^	638.50 ± 36.06^h^	2894.50 ± 64.35^de^	902.00 ± 26.87^d^	78.35 ± 1.13^bcde^
Luoshu 15	2942.00 ± 73.54^ef^	2093.50 ± 58.69^b^	848.50 ± 14.85^e^	2937.00 ± 76.37^d^	843.50 ± 17.68^ef^	75.45 ± 0.57^g^
Maximum	3579.00	2214.00	1365.00	3361.00	1152.50	81.55
Minimum	2294.00	1721.50	476.50	2404.50	683.00	75.08
Range	1285.00	492.50	888.50	956.50	469.50	6.48
Average	2882.44	1989.94	892.50	2878.89	888.94	77.65
*SD*	319.14	128.71	237.95	249.19	134.33	1.58
Variation coefficient	11.07	6.47	26.66	8.66	15.11	2.04

M ± SD. For the same index, different letters indicate significant differences with *p* < 0.05. SD, standard deviation; PV, peak viscosity; TV, trough viscosity; BD, breakdown; FV, final viscosity; SB, setback; PT, pasting temperature.

### Physicochemical properties of sweet potato starch

The physicochemical properties of all samples are presented in Table 4. The WAC and OAC of 18 kinds of sweet potato starch were significantly different. Among them, the sweet potato starch of Luoshu 11, Luoshu 14, Xushu 18, and Sushu 18 had higher WAC, ranging from 0.84 g/g to 0.87 g/g and indicating that these starches had a high concentration of hydrophilic groups. In contrast, Luxuan 1, Jishu 99, and Luoshu 14 had higher OAC, ranging from 0.70 g/g to 0.85 g/g. The results are consistent with those reported by Singh et al. (31).

**TABLE 4:** The physicochemical properties of sweet potato starch.

Variety	WAC (g/g)	OAC (g/g)	Solubility (%)	SP (%)	RR (%)	GS (kgf/cm^2^)
Yanshu 29	0.79 ± 0.02^de^	0.58 ± 0.01^hi^	7.43 ± 0.03^a^	12.42 ± 0.55^abc^	15.58 ± 3.81^g^	0.04 ± 0.00^cd^
Yushu 15	0.83 ± 0.00^abcd^	0.61 ± 0.02^fghi^	6.18 ± 1.76^ab^	12.22 ± 0.13^abc^	21.90 ± 0.61^fg^	0.05 ± 0.00^c^
Wanshu 9	0.79 ± 0.01^de^	0.60 ± 0.02^ghi^	2.48 ± 0.01^d^	12.26 ± 0.17^abc^	30.50 ± 3.25^de^	0.05 ± 0.00^c^
Luxuan 1	0.82 ± 0.04^abcd^	0.85 ± 0.07^a^	7.46 ± 3.53^a^	11.05 ± 0.69^bc^	56.23 ± 1.15^a^	0.07 ± 0.00^a^
Jishu 25	0.71 ± 0.02^f^	0.68 ± 0.00^bcd^	2.48 ± 0.01^d^	10.23 ± 0.53^c^	39.38 ± 1.01^bc^	0.05 ± 0.00^c^
Qinshu 9	0.80 ± 0.01^cde^	0.67 ± 0.01^bcde^	2.48 ± 0.02^d^	13.32 ± 0.29^abc^	19.04 ± 1.90^g^	0.03 ± 0.00^e^
Jishu 99	0.79 ± 0.02^de^	0.70 ± 0.01^bc^	2.48 ± 0.01^d^	15.00 ± 5.65^a^	34.37 ± 1.78^cd^	0.05 ± 0.00^c^
Xushu 18	0.84 ± 0.06^abc^	0.60 ± 0.00^ghi^	3.71 ± 1.75^cd^	12.65 ± 0.40^abc^	42.04 ± 4.28^b^	0.04 ± 0.00^d^
Xushu 37	0.81 ± 0.03^bcde^	0.51 ± 0.00^j^	4.94 ± 0.00^bcd^	14.33 ± 0.05^ab^	18.07 ± 1.60^g^	0.06 ± 0.00^b^
Sushu 18	0.84 ± 0.01^abcd^	0.59 ± 0.01^hi^	2.48 ± 0.01^d^	13.41 ± 0.58^abc^	18.18 ± 1.99^g^	0.05 ± 0.00^c^
Sushu 23	0.78 ± 0.02^de^	0.61 ± 0.02^fghi^	7.44 ± 0.00^a^	12.59 ± 0.49^abc^	29.37 ± 4.98^de^	0.04 ± 0.00^d^
Sushu 28	0.83 ± 0.02^abcd^	0.57 ± 0.01^i^	2.48 ± 0.00^d^	13.55 ± 0.24^abc^	31.02 ± 4.19^de^	0.07 ± 0.00^a^
Luoxushu 9	0.82 ± 0.00^abcd^	0.62 ± 0.01^efgh^	2.48 ± 0.00^d^	12.76 ± 0.48^abc^	30.07 ± 2.51^de^	0.05 ± 0.00^c^
Luoshu 10	0.75 ± 0.00^ef^	0.59 ± 0.01^hi^	4.96 ± 0.01^bc^	12.91 ± 0.11^abc^	29.74 ± 5.55^de^	0.05 ± 0.00^c^
Luoshu 11	0.87 ± 0.01^a^	0.60 ± 0.01^ghi^	2.50 ± 0.00^d^	11.52 ± 0.01^abc^	33.03 ± 3.22^cde^	0.05 ± 0.00^c^
Luoshu 12	0.82 ± 0.03^abcd^	0.65 ± 0.01^cdef^	4.98 ± 0.03^bc^	13.83 ± 0.45^ab^	26.50 ± 0.82^ef^	0.01 ± 0.00^f^
Luoshu 14	0.86 ± 0.03^ab^	0.71 ± 0.00^b^	2.48 ± 0.01^d^	11.62 ± 1.30^ab^	29.14 ± 1.14^de^	0.03 ± 0.00^e^
Luoshu 15	0.82 ± 0.01^abcd^	0.64 ± 0.01^defg^	4.96 ± 0.03^bc^	13.48 ± 0.67^abc^	17.93 ± 0.62^g^	0.03 ± 0.00^e^
Maximum	0.87	0.85	7.46	15.00	56.23	0.07
Minimum	0.71	0.51	2.48	10.23	15.58	0.01
Range	0.16	0.34	4.98	4.77	40.65	0.06
Average	0.81	0.63	4.13	12.73	29.01	0.04
*SD*	0.04	0.07	1.95	1.17	10.20	0.01
Variation coefficient	4.78	11.47	47.14	9.21	35.16	31.12

M ± SD. For the same index, different letters indicate significant differences with *p* < 0.05. SD, standard deviation; WAC, water absorption capacity; OAC, oil absorption capacity; SP, swelling power; RR, rate of retrogradation; GS, gel strength.

The solubility and SP are basic properties of starch and important properties in food processing, reflecting the interaction between starch and water. As shown in Table 4, the solubility and SP of 18 kinds of sweet potato starch were significantly different, ranging from 2.48 to 7.46% and 10.23 to 15.00%, respectively. This result is consistent with that reported by Trung et al. (32). The rate of retrogradation (RR) was related to the dehydration of starch gel. The RR of 18 kinds of sweet potato starch was significantly different, with a range of 15.58–56.23% and a coefficient of variation of 35.16%. Among them, the dewatering amount of starch gel in Luxuan 1 was significantly higher than that in other varieties, indicating that Luxuan 1 had the highest rate of regeneration. The GS of different kinds of sweet potato starch was significantly different, ranging from 0.01 to 0.07, and the coefficient of variation was 31.12%. The results are consistent with those reported by Xing et al. (15).

### Correlation analysis of quality indexes

**FIGURE 1:**
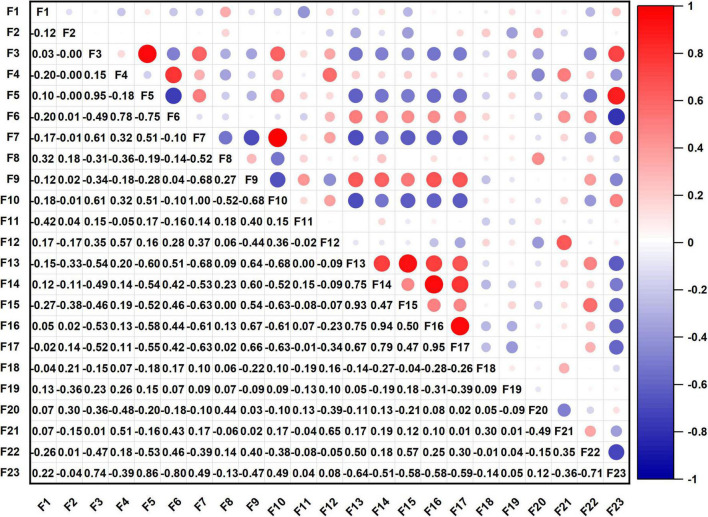
Correlation coefficient heatmap of quality indexes (F1, moisture; F2, ash; F3, total starch; F4, amylose; F5, amylopectin; F6, ratio of amylose to amylopectin; F7, L; F8, a*; F9, b*; F10, ΔE; F11, water absorption capacity; F12, oil absorption capacity; F13, peak viscosity; F14, trough viscosity; F15, breakdown; F16, final viscosity; F17, setback; F18, pasting temperature; F19, solubility; F20, swelling power; F21, rate of retrogradation; F22, gel strength; F23, sensory evaluation value).

Among the 22 components and processing quality indexes, WAC and OAC, pasting properties, S, SP, RR, and GS reflect the processing quality characteristics of sweet potato starch. As expected, there was collinearity in the measurement indexes; therefore, correlation analysis was used to determine the relationship between the indexes and provide a theoretical basis for the classification and screening of quality indexes in the next step. The heatmap of the correlation coefficient is shown in Figure 1. The circle in the upper triangular reflects correlation coefficient. The red circles represent positive correlations, and blue circles represent negative correlations. The darker (larger) the circle is, the higher the correlation coefficient is. And the specific correlation coefficients are shown in the lower triangular. Among the 231 correlations, 29 were significantly correlated at the α = 0.05 level; 25 were significantly correlated at the α = 0.01 level; and there was no significant correlation among the other 177 quality indexes. The FV was positively correlated with TV and SB, and the correlation coefficients were both 0.95. There was a significant positive correlation between total starch content and amylopectin content, and the correlation coefficient was 0.95. The PV was positively correlated with BD, and the correlation coefficient was 0.93. The results show a certain degree of independent correlation among some quality indexes, that is, information overlap existed in some indexes. Therefore, 22 indexes were needed to classify and simplify the data set to enhance the efficiency and accuracy of the quality evaluation of sweet potato starch.

### Principal component analysis of quality indexes

Principal component analysis can extract a few mutually independent indexes and reduce redundant variables (33). PCA was used to extract components from evaluation indexes based on SPSS 18.0 software, and the eigenvalue matrix of the evaluation indexes was obtained. The cumulative variance contribution rate of the first six principal components was 82.26%, and λ > 1, indicating that it could represent most of the original evaluation indexes (34, 35). The variance contribution rate indicates the dispersion degree of different index traits in the principal component. The larger the variance contribution rate is, the more important the principal component is in the analysis of sample data. The eigenvalue of the first principal component was 7.32, accounting for 33.28% of the total variance of the original 22 variables, and it was the decisive principal component. The variance contribution rate of the second principal component was 16.59%. The details are shown in Table 5.

**TABLE 5:** Eigenvalue, variance and cumulative variance contribution of principal component analysis.

Principal component	Eigenvalue	Variance contribution (%)	Cumulative variance contribution (%)
1	7.32	33.28	33.28
2	3.65	16.59	49.87
3	2.08	9.46	59.34
4	1.95	8.85	68.19
5	1.65	7.51	75.70
6	1.42	6.46	82.16

To better explain the relationship between the original evaluation indexes and principal components, the principal component load matrix was constructed by orthogonal rotation method (36, 37). The load values in Table 6 reflect the importance of each original evaluation index in the principal component. As shown in Table 6, the first principal component mainly explained three variables, namely TV, FV, and SB, and their variation coefficient dispersion was small. TV was positively correlated with FV and SB. Considering the correlation, importance, and variation coefficient of quality indexes, the SB value was selected to represent the first principal component. The second principal component corresponded with RR, amylose, and OAC. The OAC was positively correlated with RR; therefore, the RR was selected as the representative of the second principal component. The third principal component mainly integrated the information of total starch and amylopectin and AA. The total starch was positively correlated with amylopectin; AA was significantly positively correlated with amylose in the second principal component; and AA contained information about amylose and amylopectin. Therefore, the AA was selected as the representative of the third principal component. The fourth principal component mainly explained the GS; the fifth principal component mainly explained a* value; and the sixth principal component mainly explained ash and solubility. Finally, seven indexes, namely SB, RR, AA, GS, a* value, ash content, and solubility, were selected as the core indexes of the quality evaluation of sweet potato starch.

**TABLE 6:** Orthogonal rotation component matrix of the principal components.

	PC1	PC2	PC3	PC4	PC5	PC6
F1	0.14	0.10	–0.22	–0.26	0.47	0.09
F2	–0.12	–0.08	0.13	–0.06	0.13	–0.79
F3	–0.33	0.23	–0.75	–0.33	–0.21	0.10
F4	0.10	0.72	0.32	–0.15	–0.41	0.16
F5	–0.36	0.00	–0.85	–0.27	–0.08	0.04
F6	0.28	0.48	0.76	0.07	–0.24	0.08
F7	–0.55	0.29	–0.14	–0.60	–0.36	–0.03
F8	0.08	–0.03	0.05	0.16	0.94	–0.02
F9	0.65	–0.16	–0.09	0.53	0.19	–0.03
F10	–0.54	0.28	–0.14	–0.60	–0.36	–0.03
F11	0.14	0.00	–0.21	–0.08	0.19	–0.05
F12	–0.19	0.87	–0.08	–0.17	0.12	0.13
F13	0.68	0.06	0.33	0.50	–0.04	0.34
F14	0.91	0.12	0.25	0.01	0.18	0.03
F15	0.43	0.02	0.30	0.66	–0.15	0.44
F16	0.94	0.02	0.26	0.12	0.03	–0.12
F17	0.87	–0.07	0.24	0.22	–0.11	–0.26
F18	–0.53	0.22	0.41	0.20	0.15	–0.18
F19	–0.28	0.07	0.05	0.00	0.11	0.80
F20	–0.02	–0.54	0.36	–0.33	0.53	–0.15
F21	0.02	0.88	0.07	0.21	–0.01	–0.05
F22	0.01	0.21	0.29	0.79	0.03	–0.05

PC1, the first principal component; PC2, the second principal component; PC3, the third principal component; PC4, the fourth principal component; PC5, the fifth principal component; PC6, the sixth principal component. F1, moisture; F2, ash; F3, total starch; F4, amylose; F5, amylopectin; F6, ratio of amylose to amylopectin; F7, L; F8, a*; F9, b*; F10, ΔE; F11, water absorption capacity; F12, oil absorption capacity; F13, peak viscosity; F14, trough viscosity; F15, breakdown; F16, final viscosity; F17, setback; F18, pasting temperature; F19, solubility; F20, swelling power; F21, rate of retrogradation; F22, gel strength.

The original data of the seven sweet potato starch quality indexes were normalized (normalized value is X_*i*_). The results are shown in Table 7. The determination of the ideal value depended on the positive and negative directivity of the index. Solubility was a positive index, and the higher the measured value, the better the quality. Thus, the maximum value was selected as the ideal value. The a* value was a neutral index, and the average value was the ideal value. Ash content, AA, SB, RR, and GS were inverse indexes, and the minimum value was taken as the ideal value.

**TABLE 7:** Ideal value and normalized value of core quality index of sweet potato starch.

Core quality index	Ideal value (x_0_)	Normalized value (X_*i*_)			
		Minimum	Maximum	Mean	*SD*
F_2_ (%)	0.40	0.00	1.00	0.47	0.27
F_6_ (%)	26.41	0.00	1.00	0.39	0.30
F_8_	-0.17	0.00	0.99	0.73	0.24
F_17_(CP)	683.00	0.00	1.00	0.56	0.29
F_19_ (%)	7.46	0.00	1.00	0.33	0.39
F_21_ (%)	15.58	0.00	1.00	0.67	0.25
F_22_ (kgf/cm^2^)	0.01	0.00	0.93	0.44	0.23

F2, ash; F6, ratio of amylose to amylopectin; F8, a*; F17, setback; F19, solubility; F21, rate of retrogradation; F22, gel strength.

### Determination of evaluation index weight

The judgment matrix established by the 1–9 scale method is shown in Table 8. Considering the properties for brewing sweet potato starch, the importance degree of each index affecting the comprehensive evaluation system for brewing sweet potato starch was ranked. The importance ranking of indexes was as follows: AA = GS > a* value > SB = RR > ash content > solubility. The most important quality characteristic of starch was the mouthfeel of starch and starch paste. The taste and gel status of starch paste were affected by amylose content and amylopectin content; thus, the AA and GS had the greatest influence on the evaluation system. The a* value was the third most important factor affecting the evaluation of starch quality because starch color directly affects sensory perception. The SB and RR were the fourth and fifth most important factors because retrogradation affects the stability of starch and starch paste. The ash content was the sixth factor because ash represents impurities in starch. The seventh factor was solubility.

**TABLE 8:** Judgment matrix.

	F_2_ (%)	F_6_ (%)	F_8_	F_17_ (CP)	F_19_ (%)	F_21_ (%)	F_22_ (kgf/cm^2^)
F_2_ (%)	1.00	1/8	1/5	1/3	2.00	1/3	1/8
F_6_ (%)	8.00	1.00	3.00	5.00	8.00	5.00	1.00
F_8_	5.00	1/3	1.00	2.00	7.00	2.00	1/3
F_17_ (CP)	3.00	1/5	1/2	1.00	5.00	1.00	1/5
F_19_ (%)	1/2	1/8	1/7	1/5	1.00	1/5	1/8
F_21_ (%)	3.00	1/5	1/2	1.00	5.00	1.00	1/5
F_22_ (kgf/cm^2^)	8.00	1.00	3.00	5.00	8.00	5.00	1.00

F2, ash; F6, ratio of amylose to amylopectin; F8, a*; F17, setback; F19, solubility; F21, rate of retrogradation; F22, gel strength.

According to the established judgment matrix, the root method was used to test the consistency of the matrix, and the weight coefficients of each quality index were calculated. The calculated results are as follows:

*W*_1_ = 0.034, *W*_2_ = 0.321, *W*_3_ = 0.141, *W*_4_ = 0.080, *W*_5_ = 0.023, *W*_6_ = 0.080, *W*_7_ = 0.321,
AW = (0.214,2.322,1.006,0.575,0.172,0.575,2.322)*^T^*,
λ_*max*_ = 7.228, C_*I*_ = 0.038.

The mean random consistency index was queried, when n = 7, *R*_*I*_ = 1.32, *C*_*R*_ = 0.029 < 0.1. This indicates the established judgment matrix had satisfactory consistency. The weight coefficients were 0.034, 0.321, 0.141, 0.080, 0.023, 0.080, and 0.321. The determination of weight coefficients based on the method of establishing judgment matrix ensured the acquisition of reasonable and accurate evaluation results and avoided the limitations of the assignment method (36). The comprehensive evaluation model of sweet potato starch was as follows: Y (comprehensive value) = 0.034X_2_ + 0.321X_6_ + 0.141X_8_ + 0.08X_17_ + 0.023X_19_ + 0.08X_21_ + 0.321X_22_.

### Validation of quality comprehensive evaluation model of sweet potato starch

Sensory evaluation uses sensory organs to evaluate the sensory characteristics of a product and to predict the quality of the product (38, 39). Sensory evaluation was used to investigate the accuracy of the evaluation model established by the AHP. The sensory evaluation indexes of sweet potato starch mainly include color, odor, impurities of starch, and color, transparency, fluidity and consistency of starch paste. The sensory evaluation indexes and proportions were determined according to Song et al. (40, 41) (Table 9).

**TABLE 9:** Sensory evaluation indexes of sweet potato starch.

Evaluation index		Stand for evaluation	Score
Starch	Color	Uniform color, white with luster	10.5–15
		Uniform color, white	4.5–10.5
		Uniform color, dark gray	0–4.5
	Odor	With sweet potato scent	7–10
		Without sweet potato scent	3–7
		With a peculiar smell	0–3
	Impurities	No foreign impurities visible in normal vision	7–10
		A little foreign impurities can be seen with normal vision	3–7
		More foreign impurities can be seen with normal vision	0–3
Starch paste	Color	Uniform color, slightly brown with luster	10.5–15
		Uniform color, slightly brown	4.5–10.5
		Uniform color, brown	0–4.5
	Transparency	High transparency	10.5–15
		Moderate transparency	4.5–10.5
		Low transparency	0–4.5
	Fluidity	High fluidity (haw juice)	10.5–15
		Moderate fluidity (yogurt)	4.5–10.5
		Low fluidity (honey)	0–4.5
	Consistency	High consistency (honey)	14–20
		Moderate consistency (yogurt)	6–14
		Low consistency (haw juice)	0–6

**FIGURE 2:**
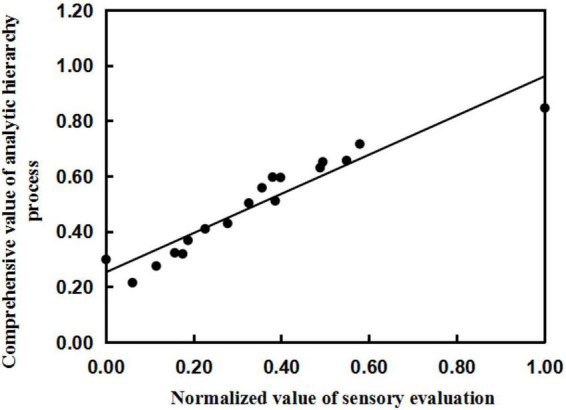
Fitness test of sensory evaluation sore and analytic hierarchy process value.

The comprehensive score of the sensory evaluation was normalized (Table 10), and the normalized value of the sensory evaluation and the comprehensive value of the AHP were fitted by regression analysis. The *x*-axis is the normalized value of the sensory evaluation, and the y-axis is the result of model calculation (comprehensive value of the AHP). The linear relationship between the two was obtained as follows: *y* = 0.708x + 0.253, *R*^2^ = 0.903 (Figure 2). The results show that the established quality evaluation model for brewing sweet potato starch can fit the normalized values of sensory evaluation and the comprehensive value of the AHP well. The results show that the comprehensive evaluation system of sweet potato starch can accurately predict the quality of sweet potato starch, indicating that it is reasonable and feasible to use the mathematical model to evaluate the quality of sweet potato starch.

**TABLE 10:** The comparison of analytic hierarchy process value and sensory evaluation score.

Quality index	Minimum	Maximum	Mean	*SD*
Comprehensive value of analytic hierarchy process	0.22	0.85	0.49	0.18
Comprehensive score of sensory evaluation	62.45	70.75	65.28	1.95
Normalized value of sensory evaluation	0.00	1.00	0.34	0.23

SD, standard deviation.

## Discussion

Sweet potato starch is one of the important processing materials, and its quality greatly affects the properties of starch processing products. There are many factors affecting the quality of sweet potato starch, including sensory quality and processing quality (42). Some of these factors are closely related and complicated to determine. Currently, there is no systematic quality evaluation system for sweet potato starch. In this study, 22 indexes of sweet potato starch were determined. The moisture content and total starch content of different sweet potato starch was similar, but the ash content, amylose and amylopectin content were significantly different. These differences affected the physicochemical properties of starch and the quality of starch products. Yong et al. found that amylose content was significantly positively correlated with enthalpy of gelatinization from purple sweet potato with different varieties (43). The study of Yu et al. found that the amylose content in sweet potato starch significantly affected the cooked weight and broken rate of starch noodles (13). The color of 18 kinds of sweet potato starch was slightly different. However, except for a* value, the variation coefficient of other color parameters was small. The RVA viscograph of 18 kinds of sweet potato starch was different. Both morphology and structural of starch and alkali concentration affected pasting properties. Liu et al. and Cardoso et al. found that the pasting properties of starch were affected by the granule size and shape, amylose and phosphorus contents, amylopectin chain length, size and molecular structure of crystallization region, and also affected the quality of starch and starch products (44, 45). The WAC of 18 kinds of sweet potato starches had small difference, while the OAC varied widely. Currently, scholars have different understandings of the differences in WAC. Yang et al. and Singh et al. believed that the high content of hydrophilic groups in starch particles, or exposure of hydrophilic groups, can cause a strong water absorption capacity (31, 46). Zhou et al. suggested that WAC was affected by the proportion of damaged starch in starch, because the water absorption capacity of damaged starch was 2.5 times that of ordinary starch (47). In addition, the WAC of starch was also affected by the size of starch particles, the tightness of structure and the relative humidity of external environment. The OAC depended on the molecular structure difference of starch (48). The solubility and SP of 18 kinds of sweet potato starch were significantly different. Upon heating of the starch paste, changes in the granule size and shape, crystallinity, degree of branching, and complexation of amylose and lipid affected the solubility and SP of starch. These changes also account for the differences between sweet potato starches. Moreover, environmental factors such as external temperature and the addition of exogenous substances also affect the solubility and swelling power of starch (49). The RR of 18 kinds of sweet potato starch was different, and was related to the amount of amylose and lipid in the starch. This was because the lipid could form a complex with amylose, which prevented the expansion of starch particles during gelatinization (50, 51). In addition, the chemical structure, molecular weight, and conformation in carbohydrate aqueous solution affected the interaction between starch molecules, thus affecting the RR of starch gel.

There is a relationship between the composition and physicochemical properties of starch. Consistent with the findings of Yu et al. (13), we found that when the total starch content was high, the amylopectin content was also higher, possibly because amylopectin was dominant in sweet potato starch, and the change of amylopectin more affected the change of total starch. The total starch content was negatively correlated with the setback value. However, Yu et al. believed that there was no obvious change rule between total starch content and setback value (13). The weak correlation between total starch content and setback value may be due to the accidental error. The AA was positively correlated with amylose content, and negatively correlated with amylopectin content. This is due to the definition of the AA. Besides, AA was positively correlated with peak viscosity. But Hou et al. found AA was negatively correlated with peak viscosity (52). The reason for the inconsistency may be that amylose and amylopectin have complex structures, and the AA is only an index to measure the ratio of amylose and amylopectin. It’s these microscopic structures that really affect the peak viscosity. When the amylose content was high, the proportion of chain A was low and the proportion of chain B1 was high, the peak viscosity of starch paste was high (11, 13, 53). Amylose content was positively correlated with OAC and negatively correlated with SP. The reason for this is that amylose forms a complex with lipid, which increases lipid absorption. Further, the complex will affect water absorbing capacity and swelling capacity in starch (49). We also noticed that amylose was positively correlated with RR, which was consistent with the study of Zhang et al. (54). Amylopectin was negatively correlated with BD and SB. This is because the cluster structure of amylopectin makes the starch chain hard to re-order and difficult to retrogradation. Therefore, when amylopectin content is high, the RR of starch is low and the starch is stable and not easy to disintegrate (55). L* value was positively correlated with ΔE. It may be that L* plays a dominant role in the calculation of ΔE, so L* greatly affects Δ*E*. The PV was positively correlated with TV, BD, FV and setback value. The TV was positively correlated with the FV and setback value. The FV was negatively correlated with the setback value. These results were consistent with the research of Hou et al. and Zhang et al. (52, 54).

In SPA, seven indexes, namely SB, RR, AA, GS, a* value, ash content, and solubility, were selected as the core indexes of the quality evaluation of sweet potato starch. The seven indexes are independent of each other. The a* was chosen as the evaluation index because a* value reflects the red and green value of sweet potato starch, which can be used to evaluate the color quality of sweet potato starch (11). The bad color of starch will affect the quality of starch and starch products, especially the quality of starch noodle. AA was chosen because AA is a basic component of starch and an important factor in determining starch properties and food functions (56). The AA affects the solubility and swelling of starch, and then affects the quality of starch noodle. The ash content was chosen because ash content is mineral and metal oxides in starch, which reflects the quality of starch. Hou et al. found that ash content was significantly positively correlated with setback value, reflecting the characteristics of starch (52). Solubility was chosen because solubility reflects the interaction between starch and water, and greatly affects the quality of starch products (20). When the solubility is small, the texture property and elasticity of starch noodle are good. The SB and RR were chosen because SB and RR reflect the recrystallization ability of starch molecules. The short-term retrogradation process of starch is due to the reorientation of dissolved amylose molecules in a parallel alignment, while long-term retrogradation is represented by the slow recrystallization of the outer branches of amylopectin (57, 58). When the RR is high, the starch paste stability is poor and the brewing ability is not good. The GS was chosen because GS refers to the firmness of the gel after the starch paste is cooled, which is one of the factors used to evaluate the quality of starch. The shorter the recrystallization time during starch aging is, the greater the GS is (20). Sweet potato starch with high gel strength is suitable for making jelly and other products that have certain requirements on gel strength.

Evaluation model of sweet potato starch is the core part of evaluation system. The *R*^2^ of the sweet potato starch model established in this study reached 0.903. The results showed that the starch quality could be predicted well. Compared with the existing evaluation methods of sweet potato starch, the evaluation method established in this study integrated all the core evaluation indexes, and scientifically assigned weights to each index. So far, there is no systematic quality evaluation method for sweet potato starch, however, similar methods have been applied to the evaluation of fresh fruit. Bai et al. took different varieties of apples as the research objects, measured the quality indexes of fresh apples, analyzed the distribution of indexes and the correlation among indexes, screened eight evaluation indexes such as SSC, and finally established the comprehensive quality evaluation method of fresh apples by objective assignment method (59). Nie et al. (60) established a quality evaluation model for apple juice. He measured the quality indexes of Fresh apple juice, screened the evaluation indexes of fresh apple juice by factor analysis, decided the weight by analytic hierarchy process, and established discrimination functions of fresh apple juice quality by K-means cluster and discriminant analysis. The established discriminant function has high accuracy and can be used to distinguish the quality of apple juice (60). The accuracy of the quality evaluation model of sweet potato starch is similar to that of the fresh fruit model, which indicates that it is reasonable and feasible to establish sweet potato starch quality evaluation model based on multivariate statistical analysis method. In this study, we expanded the research object of the quality evaluation model from fresh food to processed products, which provided a new idea and method for the evaluation of processed products.

## Conclusion

A quality evaluation system of brewing sweet potato starch was established based on a multivariate statistical analysis method. There were different degrees of correlation among different evaluation indexes. Seven core indexes, namely SB, RR, AA, GS, a* value, ash content, and solubility, were screened by PCA. The quality evaluation model of sweet potato starch was *Y* = 0.034X_2_ + 0.321X_6_ + 0.141X_8_ + 0.08X_17_ + 0.023X_19_ + 0.08X_21_ + 0.321X_22_. The comprehensive quality evaluation system of sweet potato starch established in this experiment is of great significance for the post-harvest processing of high-starch sweet potato. The results provide a new idea for the quality evaluation of sweet potato starch and its products.

## Data availability statement

The original contributions presented in this study are included in the article/supplementary material, further inquiries can be directed to the corresponding authors.

## Author contributions

CM: investigation, formal analysis, writing – original draft, writing – review and editing. WZ: chemical analysis. YZ and RY: data analysis. YL, ZM, and HZ: contributed reagent, analysis tools, and materials. JS and FN: conceptualization, methodology, and supervision. All authors contributed to the article and approved the submitted version.

## Abbreviations

PCA: principal component analysis
AHP: analytic hierarchy process
AA: ratio of amylose to amylopectin
WAC: water absorption capacity
OAC: oil absorption capacity
PV: peak viscosity
TV: trough viscosity
FV: final viscosity
BD: breakdown
SB: setback
PT: pasting temperature
SP: swelling power
RR: rate of retrogradation
GS: gel strength.
